# Trends and determinants of complete vaccination coverage among children aged 12–59 months: An analysis of Bénin Demographic and Health Surveys from 1996 to 2018

**DOI:** 10.1371/journal.pgph.0004206

**Published:** 2025-02-20

**Authors:** Jean-Pierre Gnimatin, Shiméa M. Agossou, Lauryn L. A. Hinde, Joyce Aputere Ndago, Emmanuel Owusu Dankwah, Joël Segnon, Quynh Ngoc Thuy Ho, Martin Nyaaba Adokiya

**Affiliations:** 1 School of Public Health, University for Development Studies, Tamale, Ghana; 2 Department of Epidemiology, Regional Institute of Public Health, University of Abomey-Calavi, Ouidah, Bénin; 3 UFR Santé, University of Caen Normandy, Caen, France; 4 UFR Sciences et Techniques, University of Rouen Normandy, Rouen, France; 5 School of Public Health, University of Ghana, Ghana; 6 Pham Ngoc Thach University of Medicine, Vietnam; VART Consulting PVT LTD, INDIA

## Abstract

Vaccination is pivotal for global public health, yet achieving complete coverage among children in low-income countries remains challenging. This study assessed vaccination trends in children aged 12–59 months using Demographic and Health Surveys (DHS) data from 1996 to 2018 in Bénin. The study incorporated a range of independent variables sourced from prior studies. The data was processed and analyzed using R version 4.2.0, employing a combination of inferential and descriptive statistical techniques. Both univariate and multivariable binary logistic regression analyses were conducted to explore the determinants of complete vaccination coverage. The trend of complete childhood vaccination coverage in Bénin has shown fluctuations, with rates increasing from 47% in 1996 to 55% in 2017–2018. Higher levels of parental education -fathers (aOR 1.41; 95% CI 1.15–1.73) and mothers (aOR 1.69; CI 1.12–2.57), and urban residence (aOR 1.08; CI 1.00–1.16), were associated with complete childhood vaccination coverage. This association was also found for other factors such as antenatal care visits (aOR 1.15; CI 1.04–1.28) and deliveries at healthcare facilities (aOR 2.48; CI 2.22–2.77). Despite significant progress overtime, challenges persist, particularly among younger and rural mothers. Targeted interventions, like community-based vaccination advocacy and effective reminder systems, are essential to addressing these issues and improving vaccination coverage.

## Introduction

Vaccination is widely recognized as one of the most cost-effective public health interventions, playing a pivotal role in preventing morbidity, mortality, and disability from a range of infectious diseases [1,2]. Ensuring complete vaccination coverage among children is a critical component of global efforts to achieve universal health coverage and improve child survival rates [3]. The under-five mortality rate has dropped by 59% at the global level from 93 deaths per 1,000 live births in 1990 to 37 deaths per 1,000 live births in 2022 [4]. Most under-five deaths are caused by diseases that are readily preventable or treatable with proven, cost-effective interventions [4]. Among the available interventions, vaccination stands out as a pivotal tool for averting life-threatening infectious diseases [1]. Findings from 98 low- and middle-income countries (LMICs) revealed that many deaths were averted through vaccination per calendar year. An estimated 69 million (52–88) deaths will have been prevented between 2000 and 2030, including 37 million (30–48) between 2000 and 2019 [2].

Despite the existing health and economic advantages of childhood vaccinations, there are significant uptake disparities in poor-income countries [5]. UNICEF revealed that over a span of three years (2019–2021), 12.7 million children in Africa did not receive one or more vaccinations. Globally, the total number of children missing out on vaccinations between 2019 and 2021 amounted to 67 million, and vaccination coverage levels declined in 112 countries [6]. In view of these disparities, the increased number of children who are susceptible to vaccine-preventable diseases (VPDs) suggests a high risk of VPDs outbreaks in the future [7]. This threat might have negative consequences for households, communities, and national economies. Recurrent and serious illnesses put a burden on financial resources, healthcare, and general well-being of populations [8]. It is therefore important to monitor and understand vaccination coverage in developing countries to enable ongoing policy decision-making that increases and maintains coverage.

The childhood vaccination coverage in Bénin, a country in west Africa, presents a sobering reality. Despite commendable strides, the health status of children remains a cause for concern [9]. From 2000 to 2019, three Vaccine-Preventable Diseases (VPDs) have persistently ranked among the top 10 causes of death among children under five in Bénin [10]. Specifically, in 2019, the death rates per 100,000 population among children aged 0 to 1 year in Bénin were as follows: 149.54 for Meningitis, 136.86 for whooping cough (pertussis), and 86.01 for Measles. Among children aged 1 to 4 years, the corresponding rates were 19.38 for Meningitis, 33.91 for whooping cough (pertussis), and 24.14 for Measles [10]. In Bénin, the mortality rate for children under 5 dropped from 98 to 84 per 1,000 live births between 2015 and 2021. This remains above the Sustainable Development Goal (SDG) target of 25 per 1,000 live births. Similarly, the neonatal mortality rate fell from 32 to 29 per 1,000 live births over the same period. This also exceeds the target of 12 per 1,000 set by the SDGs [11]. With VPDs being continuously ranked among the top 10 causes of death in the country [10], it has become important to explore vaccination rates among children in the country. To provide evidence-based insights for policy-makers, public health practitioners, and stakeholders in vaccination programs; this study aims to conduct a comprehensive analysis of the trends and determinants of complete vaccination coverage among children aged 12–59 months using data from the Bénin Demographic and Health Surveys conducted between 1996 and 2018. Understanding the contribution of these factors is essential for formulating targeted interventions to further increase complete vaccination coverage for improving child health outcomes in Bénin and beyond.

## Methodology

### Study setting

Bénin is situated in West Africa and lies within the tropical zone between the equator and the Tropic of Cancer. It spans an area of 114,763 square kilometers. As of 2016, Bénin’s population was 10,741,458. The nation shares borders with Togo to the west, Nigeria to the east, Niger to the northeast, and Burkina Faso to the northwest [12]. While Bénin has seen notable economic growth in recent years, achieving lasting poverty reduction remains a significant challenge. The nation is among the world’s least developed countries, with a per capita income of $770 in 2016. It was placed at 163^rd^ out of 188 countries on the Human Development Index 2017 with a score of 0.515 [13]. Administratively, Bénin is divided into 12 departments. These are: Atacora, Donga, Borgou, Alibori, Atlantique, Littoral, Mono, Couffo, Ouémé, Plateau, Zou and Collines [12].

### Study type and data sources

The present study adopts a quantitative approach characterized by a retrospective and analytical design. A secondary analysis of data was performed utilizing information derived from five consecutive Bénin Demographic and Health Surveys (BDHS) conducted between 1996 and 2018. These surveys, carried out at five-year intervals, aimed to provide estimates of fundamental demographic and health metrics within the nation. To gain access to the BDHS data, a research concept note was formulated and submitted through the Demographic and Health Surveys (DHS) program website at http://www.dhsprogram.com. This process entailed providing a concise overview of the planned study, including research objectives, variables of interest, and the intended timeframe. Subsequently, approval was obtained from the Demographic and Health Service (DHS) program for access to the requested datasets.

### Data source and study population

The BDHS employed a two-stage, stratified cluster design, encompassing urban and rural areas. In the initial stage, clusters were chosen from a comprehensive list of enumeration areas. Subsequently, households were allocated to each selected cluster in the second stage [14]. In all the BDHS studies, the response rate ranged from 94% to 98%. From there, households were systematically chosen to participate in the survey, focusing on women aged 15–49 and those with children aged 12–59 months (at the time of the survey). In total, 17,294 children under five years old were included in the analyses (BDHS1996 = 1491; BDHS2001 = 1927; BDHS2006 = 5588; BDHS2011-12 = 4864; BDHS2017-18 = 3424).

In the Bénin Demographic and Health Surveys, all women between the ages of 15 and 49 residing in the selected households were eligible for participation in the interview, which involved the administration of the Women’s Health Questionnaire. The survey encompassed a wide range of topics, including demographic information (e.g. age, education level, media exposure), reproductive history (births and childhood mortality), family planning practices, fertility preferences, antenatal and postnatal care, breastfeeding and infant feeding habits, childhood vaccination and illness history, marital status and sexual activity, women’s employment, husband’s background characteristics, awareness and behaviors related to Human Immunodeficiency Virus/Acquired Immunodeficiency Syndrome (HIV/AIDS), and other sexually transmitted infections, adult mortality, including maternal mortality, and knowledge of various health issues, including tuberculosis. For the purposes of this study, we focused exclusively on extracting data related to background characteristics, maternal health, and child health.

### Study variables

#### Dependent variable.

The dependent/outcome variable for this study was complete vaccination coverage among children 12–59 months. Children were considered to have complete vaccination coverage if they have received at least: One dose of BCG vaccine, which protects against tuberculosis, Three doses of DTP vaccine (DTP1-3), which protects against diphtheria, tetanus, and whooping cough; One dose of Oral Polio Vaccine given at birth (OPV0); Three other doses of Oral Polio Vaccine (OPV1-3); and One dose of measles vaccine (MMR) according to vaccination card or mother’s declaration, as per the survey’s established methodology and data collection protocols [15,16].

#### Independent variables.

The study incorporated a range of independent variables sourced from prior studies [17]. These variables encompassed the following aspects: Year (of the survey), age/age group of respondents, department, educational level, residence area, marital status, ethnicity, place of residence, religion, employment status (of respondents), number of children, child’s age (in months) and gender, attendance of antenatal care during pregnancy, as well as the place of delivery. The age groups of respondents were stratified into 15–19, 20–24, 25–29, 30–34, 35–39, 40–44, and 45–49. Departments were delineated as Atacora, Donga, Borgou, Alibori, Atlantique, Littoral, Mono, Couffo, Ouémé, Plateau, Zou, and Collines. Educational levels were categorized as no education, primary, secondary, and higher. The place of residence was coded as either rural or urban. Marital status was recategorized as married, never married, widowed, divorced, and cohabitating. The term “number of children” refers to the count of children a woman has given birth to, with the youngest child being the focal point for complete vaccination coverage in this study. Responses were grouped into one birth, two to three births, four to five births, and six or more births. Employment status was redefined as either working or not working. Ethnicity was reclassified as Adja, Bariba, Betamaribe, Dendi, Fon, Peulh, Yoa-Lokpa, Yoruba, Other ethnicities, and Foreigner. Religion was redefined as Traditionalist (Vodoun), Christianity, Islam, other religions, and no religion.

### Statistical analysis

The data was processed and analyzed using R version 4.2.0, employing a combination of inferential and descriptive statistical techniques. Records with missing values were excluded. Initially, descriptive analysis was employed to establish the frequency and proportion of dependent variables. Following this, a graphical representation illustrating the trends in complete vaccination percentages from 1996 to 2017–2018 was generated. Subsequently, both univariate and multivariable binary logistic regression analyses were conducted to explore the determinants of complete vaccination coverage. In the univariate analysis, variables with a P-value below 0.2 were selected for the initial multivariable logistic regression model. In this study, we employed the Akaike Information Criterion (AIC) as a criterion for model selection. The StepAIC function was applied to refine the model, resulting in the final multivariable logistic regression model. It is important to note that the absolute value of AIC does not hold intrinsic significance; instead, the focus is on comparing AIC values across different models. A model with a lower AIC value is preferred, indicating a better fit. Thus, it was also assessed whether AIC values increase or decrease as we add more variables to the model [18]. Significance was determined with a P-value of less than 0.05 in the regression models. Crude odds ratios (cORs) and adjusted odds ratios (aORs) along with their corresponding 95% confidence intervals (CIs) were calculated to measure the association between the independent variables and vaccination completion. For the regression analyses we removed observations with missing values.

### Ethical consideration

This study relies on openly accessible datasets sourced from the DHS repository (http://www.dhsprogram.com), ensuring the removal of any identifiable information. Each study respondent provided written informed consent before participation in surveys. All reports are available through the following link: The DHS Program - Country Bénin. The Institutional Review Board (IRB)-approved procedures for DHS public-use datasets do not in any way allow respondents, households, or sample communities to be identified. There are no names of individuals or household addresses in the data files. Given the complete anonymity of the data, the authors proceeded without the necessity for additional ethical approval. Nevertheless, the authors diligently completed the registration process and obtained permission to download and utilize the datasets.

## Results

### Socio-demographic characteristics per vaccination completeness status

In this study, we investigated the trends and determinants of complete vaccination coverage of children aged 12–59 months in Bénin. Our analysis relied on data gathered from five consecutive Bénin Demographic and Health Surveys conducted between 1996 and 2017–2018. The median age of the mothers was 29 years. About 29.21% and 22.68% of the mothers were 25–29 years and 30–34 years respectively. The highest rates of complete child vaccination coverage (45.72%) were among women aged 25–29 (n = 2,310), closely followed by the 20–24 age group at 45.71% (n = 1,459). Generally, 58.50% of children born to mothers in the 15–19 age group lacked complete vaccination coverage. In this study, 83.1% of the mothers in our study were married. The prevailing religions among the participants were Christianity (48.16%), Islam (26.16%), and Traditionalist (Vodoun) (17.38%). Out of the 12 departments, four revealed complete vaccination coverage of more than 50%: Littoral (56.08%), Donga (52.83%), Collines (52.56%), and Oueme (50.93%). The majority of survey respondents resided in rural areas (65.58%), and the most prevalent ethnicity was Fon (38.31%). Educational attainment among the mothers varied, with 73.81% having received no formal education, while only 0.73% had attained higher education levels. In contrast, 57.59% of the children’s fathers had no formal education, and 3.55% had achieved higher education (Table 1).

**Table 1:** Distribution of socio-demographic characteristics per the vaccination completeness status.

Characteristics	Total N = 17,294 (100%)	Complete, N = 7,704 (44.50%)	Incomplete N = 9,590 (55.50%)
**Mother’s age group**			
15–19	598 (3.50%)	248 (41.50%)	350 (58.50%)
20–24	3,192 (18.46%)	1,459 (45.71%)	1,733 (54.29%)
25–29	5,052 (29.21%)	2,310 (45.72%)	2,742 (54.28%)
30–34	3,923 (22.68%)	1,716 (43.74%)	2,207 (56.26%)
35–39	2,724 (15.75%)	1,193 (43.80%)	1,531 (56.20%)
40–44	1,319 (7.63%)	573 (43.44%)	746 (56.56%)
45–49	486 (2.81%)	205 (42.18%)	281 (57.82%)
**Marital status**			
Cohabiting	2,516 (14.55%)	1,143 (45.43%)	1,373 (54.57%)
Never married	104 (0.60%)	38 (36.54%)	66 (63.46%)
Married	14,363 (83.05%)	6,410 (44.63%)	7,953 (55.37%)
Divorced	153 (0.89%)	50 (32.68%)	103 (67.32%)
Widowed	158 (0.91%)	63 (39.87%)	95 (60.13%)
**Religion**			
Christianism	8,328 (48.16%)	4,154 (49.88%)	4,174 (50.12%)
Traditionalist (Vodoun)	3,006 (17.38%)	1,094 (36.39%)	1,912 (63.61%)
Islam	4,524 (26.16%)	1,897 (41.93%)	2,627 (58.07%)
Other religions	263 (1.52%)	113 (42.97%)	150 (57.03%)
No religion	1,173 (6.78%)	446.00 (38.02%)	727.00 (61.98%)
**Department**			
Alibori	1,223 (7.07%)	425 (34.75%)	798 (65.25%)
Atacora	1,897 (10.97%)	875 (46.13%)	1,022 (53.87%)
Atlantique	2,048 (11.84%)	879 (42.92%)	1,169 (57.08%)
Borgou	2,090 (12.09%)	835 (39.95%)	1,255 (60.05%)
Collines	1,075 (6.22%)	565 (52.56%)	510 (47.44%)
Couffo	1,111 (6.42%)	337 (30.33%)	774 (69.67%)
Donga	848 (4.90%)	448 (52.83%)	400 (47.17%)
Littoral	913 (5.28%)	512 (56.08%)	401 (43.92%)
Mono	1,409 (8.15%)	636 (45.14%)	773 (54.86%)
Oueme	1,936 (11.19%)	986 (50.93%)	950 (49.07%)
Plateau	819 (4.74%)	245 (29.91%)	574 (70.09%)
Zou	1,925 (11.13%)	961 (49.92%)	964 (50.08%)
**Residence**			
Rural	11,342 (65.58%)	4,720 (41.62%)	6,622 (58.38%)
Urban	5,952 (34.42%)	2,984 (50.13%)	2,968 (49.87%)
**Mother’s education level**			
No education	12,764 (73.81%)	5,180 (40.58%)	7,584 (59.42%)
Primary	2,871 (16.60%)	1,512 (52.66%)	1,359 (47.34%)
Secondary	1,532 (8.86%)	929 (60.64%)	603 (39.36%)
Higher	127 (0.73%)	83 (65.35%)	44 (34.65%)
**Ethnicity**			
Adja	2,768 (16.01%)	1,090 (39.38%)	1,678 (60.62%)
Bariba	1,789 (10.34%)	867 (48.46%)	922 (51.54%)
Betamaribe	1,278 (7.39%)	608 (47.57%)	670 (52.43%)
Dendi	599 (3.46%)	232 (38.73%)	367 (61.27%)
Fon	6,625 (38.31%)	3,204 (48.36%)	3,421 (51.64%)
Peulh	1,177 (6.81%)	238 (20.22%)	939 (79.78%)
Yoa-Lokpa	790 (4.57%)	390 (49.37%)	400 (50.63%)
Yoruba	1,822 (10.54%)	874 (47.97%)	948 (52.03%)
Other ethnies	34 (0.20%)	13 (38.24%)	21 (61.76%)
Foreigner	412 (2.38%)	188 (45.63%)	224 (54.37%)
**Employment status**			
Not Working	2,262 (13.08%)	811 (35.85%)	1,451 (64.15%)
Work	15,032 (86.92%)	6,893 (45.86%)	8,139 (54.14%)
**Father’s education level**			
No education	9,959 (57.59%)	3,844 (38.60%)	6,115 (61.40%)
Primary	3,812 (22.04%)	1,893 (49.66%)	1,919 (50.34%)
Secondary	2,909 (16.82%)	1,579 (54.28%)	1,330 (45.72%)
Higher	614 (3.55%)	388 (63.19%)	226 (36.81%)
**Child’s gender**			
Female	8,567 (49.54%)	3,792 (44.26%)	4,775 (55.74%)
Male	8,727 (50.46%)	3,912 (44.83%)	4,815 (55.17%)
**ANC visits**			
No	2,235 (12.92%)	265 (11.86%)	1,970 (88.14%)
1–3	4,907 (28.38%)	2,241 (45.67%)	2,666 (54.33%)
4–6	7,193 (41.59%)	3,605 (50.12%)	3,588 (49.88%)
More than 6	2,959 (17.11%)	1,593 (53.84%)	1,366 (46.16%)
**Number of children**			
1	2,803 (16.21%)	1,365 (48.70%)	1,438 (51.30%)
2–3	5,977 (34.56%)	2,722 (45.54%)	3,255 (54.46%)
4–5	4,557 (26.35%)	1,986 (43.58%)	2,571 (56.42%)
6+	3,957 (22.88%)	1,631 (41.22%)	2,326 (58.78%)
**Place of delivery**			
Home	3,437 (19.87%)	688 (20.02%)	2,749 (79.98%)
Health care setting	13,857 (80.13%)	7,016 (50.63%)	6,841(49.37%)

A significant segment of the study’s participants consisted of working mothers, representing 86.92% (n = 15,032). Among this group of working mothers, 45.86% confirmed that their children had received complete vaccination coverage. In terms of antenatal care (ANC) visits, 41.59% of the mothers had 4–6 visits, while 28.38% indicated 1–3 visits. Notably, 12.92% of mothers did not have any ANC visits. This subset revealed a high rate of incomplete child vaccination coverage (88.14%). Furthermore, the majority (34.56%) of the mothers reported that they had 2–3 children. In this study, the most prevalent place of delivery was a healthcare setting (80.13%) of all cases.

### Trends of complete vaccination coverage among children

**Fig 1:**
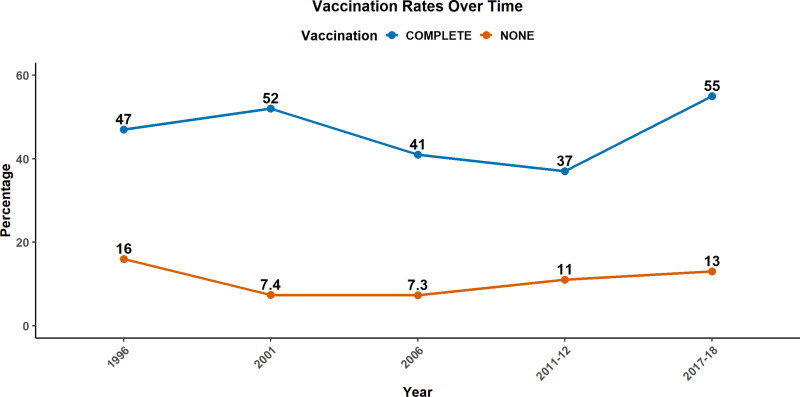
Trends of complete vaccination coverage among children aged 12 to 56 months.

The trends of complete vaccination coverage among children from 1996 to 2017–2018 are presented in Fig 1. In terms of complete vaccination coverage, there was an initial increase from 47% in 1996 to 52% in 2001. Subsequently, there was a consecutive decline to 41% in 2006 and further to 37% in 2011–2012. However, a significant upturn occurred in 2017–2018, reaching 55%. Regarding children who received no vaccines, the percentage decreased from 16% in 1996 to 7.3% in 2006. However, a subsequent rise was observed, reaching 13% in 2017–2018.

### Determinants of complete childhood vaccination coverage

The univariate analyses revealed that the age and marital status of mothers as well as the child’s gender were not significant determinants of complete vaccination coverage among children in this study. However, the department of residence of mothers was significant. The number of children although included in our initial multivariable model, was subsequently found not to be significant. Variations were found among different departments, with odds ratios indicating the likelihood of complete vaccination. Children whose mothers were residents of an urban area had 1.08 (aOR: 1.08; CI: 1.00–1.16) more odds of having complete vaccination compared to those in rural areas respectively in multivariate analyses. Higher education levels of fathers (aOR:1.41; CI: 1.15–1.73) and mothers (aOR: 1.69; CI: 1.12–2.57) were associated with complete vaccination coverage of children. The number of ANC visits is positively associated with vaccination coverage. Children whose mothers had more than 6 ANC visits had 1.15 times more odds of being completely vaccinated (aOR: 1.15; CI: 1.04–1.28). meanwhile, among those whose mothers had no ANC visits, the odds were lowered (aOR: 0.26; CI: 0.22–0.30). Additionally, the findings revealed that delivery in healthcare settings increased by 2.48, the odds of complete vaccination. That is, the odds of complete vaccination coverage among children compared to those who were given birth at home (aOR: 2.48; CI: 2.22–2.77). Tables 2 and 3 respectively present the results of univariate and multivariable logistic regression analyses with odds ratios (OR), 95% confidence intervals (CI), and corresponding p-values (used to assess the statistical significance of the coefficients associated with each predictor variable in the model) for various factors related to childhood vaccination. The final multivariable regression model had a fair performance with an area under the curve (AUC) of 0.68 (S1 Fig).

**Table 2:** Univariate logistic regression analysis of demographic characteristics associated with complete vaccination coverage.

Characteristic	cOR^*1,2*^	95% CI^*2*^	p-value
**Mother’s age group**			
25–34	—	—	
15–24	1.01	0.93, 1.09	0.90
35–49	0.95	0.88, 1.02	0.14
**Department**			
Alibori	—	—	
Atacora	1.61***	1.39, 1.87	<0.001
Atlantique	1.41***	1.22, 1.64	<0.001
Borgou	1.25**	1.08, 1.45	0.003
Collines	2.08***	1.76, 2.46	<0.001
Couffo	0.82*	0.69, 0.97	0.023
Donga	2.10***	1.76, 2.52	<0.001
Littoral	2.40***	2.01, 2.86	<0.001
Mono	1.54***	1.32, 1.81	<0.001
Oueme	1.95***	1.68, 2.26	<0.001
Plateau	0.80*	0.66, 0.97	0.023
Zou	1.87***	1.62, 2.17	<0.001
**Residence**			
Rural	—	—	
Urban	1.41***	1.32, 1.50	<0.001
**Mother’s education level**			
No education	—	—	
Primary	1.63***	1.50, 1.77	<0.001
Secondary	2.26***	2.02, 2.51	<0.001
Higher	2.76***	1.92, 4.02	<0.001
**Marital status**			
Never married	—	—	
Cohabiting	1.45	0.97, 2.19	0.076
Married	1.40	0.94, 2.11	0.10
Divorced	0.84	0.50, 1.43	0.5
Widowed	1.15	0.69, 1.93	0.6
**Work**			
Not Working	—	—	
Work	1.52***	1.38, 1.66	<0.001
**Father’s education level**			
No education	—	—	
Primary	1.57***	1.46, 1.69	<0.001
Secondary	1.89***	1.74, 2.05	<0.001
Higher	2.73***	2.31, 3.24	<0.001
**Number of children**			
2–3	—	—	
1	1.14**	1.04, 1.24	0.006
4–5	0.92*	0.85, 1.00	0.045
6+	0.84***	0.77, 0.91	<0.001
**Child’s gender**			
Female	—	—	
Male	1.02	0.96, 1.09	0.5
**ANC visits**			
1–3	—	—	
No	0.16***	0.14, 0.18	<0.001
4–6	1.20***	1.11, 1.29	<0.001
More than 6	1.39***	1.27, 1.52	<0.001
**Place of delivery**			
Home	—	—	
Health care setting	4.10***	3.75, 4.49	<0.001

* p < 0.05;

** p < 0.01;

*** p < 0.001; COR = Crude Odds Ratio, CI = Confidence Interval.

**Table 3:** Multivariable logistic regression analysis of characteristics associated with complete vaccination coverage.

Characteristic	aOR^*1,2*^	95% CI^*2*^	p-value
**Department**			
ALIBORI	—	—	
ATACORA	1.51***	1.28, 1.78	<0.001
ATLANTIQUE	0.63***	0.54, 0.74	<0.001
BORGOU	1.22*	1.03, 1.43	0.018
COLLINES	1.07	0.89, 1.28	0.5
COUFFO	0.46***	0.38, 0.56	<0.001
DONGA	1.67***	1.37, 2.03	<0.001
LITTORAL	0.80*	0.66, 0.98	0.034
MONO	0.93	0.78, 1.11	0.4
OUEME	0.88	0.75, 1.04	0.14
PLATEAU	0.46***	0.37, 0.56	<0.001
ZOU	0.92	0.79, 1.09	0.3
**Residence**			
Rural	—	—	
Urban	1.08*	1.00, 1.16	0.042
**Mother’s education level**			
No education	—	—	
Primary	1.18***	1.08, 1.30	<0.001
Secondary	1.50***	1.32, 1.71	<0.001
Higher	1.69*	1.12, 2.57	0.013
**Work**			
Not Working	—	—	
Work	1.48***	1.34, 1.64	<0.001
**Father’s education level**			
No education	—	—	
Primary	1.18***	1.08, 1.28	<0.001
Secondary	1.19***	1.08, 1.31	<0.001
Higher	1.41**	1.15, 1.73	0.001
**ANC visits**			
1–3	—	—	
No	0.26***	0.22, 0.30	<0.001
4–6	1.08*	1.00, 1.17	0.046
More than 6	1.15**	1.04, 1.28	0.006
**Place of delivery**			
Home	—	—	
Health care setting	2.48***	2.22, 2.77	<0.001

* p < 0.05;

** p < 0.01;

*** p < 0.001; AOR = Adjusted Odds Ratio, CI = Confidence Interval.

## Discussion

This study assessed the trends and determinants of complete vaccination coverage. It focused on women aged 15–49 who had children aged 12–59 months (at the time of the survey). In total, 17,294 children under five years old were included in the analyses (BDHS1996 = 1491; BDHS2001 = 1927; BDHS2006 = 5588; BDHS2011-12 = 4864; BDHS2017-18 = 3424) after removing missing and extraneous observations. The study revealed that though age was not a significant factor associated with complete vaccination, the highest complete vaccination coverages were observed among children whose mothers belonged to the age groups of 20–24 (45.71%), and 25–29 (45.72%). This aligns with observations from Ghana, which is another West African nation, where data spanning from Demographic and Health Surveys conducted between 1998 and 2014 was used for their study. Their findings also highlighted that children of mothers belonging to these age groups were among those with the highest proportions of complete vaccination coverage [19]. Mothers in these age groups may be more likely to have regular interactions with healthcare systems due to their childbearing stage. This increased engagement could enhance their awareness of the importance of childhood vaccinations and the availability of vaccination programs [20]. Additionally, it has been reported that mothers understanding/knowledge, attitudes, and practices can have a significant impact on decisions like those involving a child’s vaccines [21]. About 58.53% of children born to mothers aged 15–19 years had incomplete vaccination coverage, making it the highest among all age groups. Several factors may contribute to this phenomenon. Firstly, these mothers are still teenagers and many of them may be grappling with the demands of furthering their education or securing employment coupled with the responsibilities of raising a child [22]. The complex interplay of these commitments can potentially lead to challenges in their healthcare services utilization for themselves and their children [23,24]. Moreover, existing literature in Sub-Saharan Africa indicates that socioeconomic factors serve as barriers to the optimal vaccination of children [25]. Our study reported vaccination levels at 37% in 2011–2012 and 55% in 2017–2018. This corroborates findings from previous independent studies where population data from Bénin showed regional profiles. They found complete vaccination coverage to be at 35% in 2011–2012 and 58.1% in 2018. The slight differences observed could be explained by the fact that our study focused on children aged 12–59 months while theirs only included children between 12 and 23 months [26,27]. Though from the recent data available there has been a rise in the coverage, more efforts are needed to increase the coverage and reduce the proportion of no-vaccine uptake. Our results revealed an increase in complete vaccination coverage, from 47% in 1996 to 55% in 2017–2018. This positive progression in complete vaccination coverage trends reflect concerted efforts by various organizations and the government, to strengthen immunization programs. These initiatives include the increased allocation of financial resources and the implementation of intensive vaccination campaigns aimed at extending the reach of childhood immunization services [28–30].

Educational attainment among the mothers varied, with 73.81% having received no formal education. However, higher education levels of both fathers (aOR:1.41; CI: 1.15–1.73) and mothers (aOR: 1.69; CI: 1.12–2.57) were associated with complete vaccination coverage of children. Similar observations were reported from a pooled data analysis from Gambia, Liberia, and Sierra Leone which investigated the determinants of childhood vaccination status among children aged 0–12 months. These associations in the previous studies may be due to several factors. Parents with higher education levels are likely to have better access to information about the importance of vaccinations and the potential risks associated with vaccine-preventable diseases [31]. They may be more informed about healthcare services, including the availability and benefits of routine childhood vaccinations [32,33]. Additionally, higher education levels often correlate with improved socio-economic status and parents with higher education are more likely to have stable employment and higher incomes, which can facilitate better access to healthcare services, including vaccination programs [34].

In univariate and multivariable analyses, there were notable differences observed across various departments. These differences were reflected in odds ratios, which indicate the likelihood of achieving complete childhood vaccination coverage in each department. In univariate and multivariate analyses, children whose mothers were residents of an urban area had 1.41 (cOR:1.41; CI: 1.32–1.50) and 1.08 (aOR: 1.08; CI:1.00–1.16) more odds of having complete vaccination compared to those in rural areas respectively. Variations in complete vaccination coverage due to the spatial location have also been reported in previous studies from Ghana, and 12 East African countries (Burundi, Ethiopia, Comoros, Uganda, Rwanda, Tanzania, Mozambique, Madagascar, Zimbabwe, Kenya, Zambia, and Malawi) [19,35,36]. The same applies to Peru and Nepal, in South America and South Asia respectively [37,38]. All these studies revealed that vaccination coverage levels were generally higher in urban areas. Discrepancies in healthcare infrastructure among departments may play a pivotal role. Departments with well-established healthcare facilities and efficient service delivery systems may exhibit higher vaccination rates compared to those with limited access to quality healthcare services. Regions with lower socioeconomic status often face challenges related to accessibility, awareness, and affordability of vaccination services, impacting the odds of complete childhood vaccination [22]. Urban areas typically boast better infrastructure, more healthcare facilities, and improved accessibility to vaccination centers, which can positively influence vaccination rates [39]. However, rural areas may face challenges such as limited healthcare infrastructure, longer travel distances to reach vaccination sites, and difficulties in maintaining a cold chain for vaccine storage, all of which could contribute to lower vaccination rates [39]. Furthermore, cultural beliefs and practices might influence vaccination decisions differently in urban and rural settings [40]. Urban areas may experience greater exposure to health promotion campaigns, leading to a more positive perception of vaccinations. Conversely, rural communities might hold traditional beliefs or misconceptions that contribute to vaccine hesitancy or lower acceptance [40].

Our study highlights the crucial influence of antenatal care (ANC) visits on vaccination coverage. A positive association links the number of ANC visits to higher odds of children achieving complete childhood vaccination coverage. Additionally, healthcare facility deliveries substantially increased the odds of complete childhood vaccination coverage by 2.48 times compared to home births These results underscore the interconnected impact of ANC practices and delivery settings on childhood vaccination outcomes. These associations have been equally reported in other studies [41,42]. These could be explained by the fact that mothers who attend more ANC visits might have heightened awareness of the importance of vaccinations for their child’s health. In fact, the regular interactions with healthcare providers during ANC visits provide opportunities for education on vaccination [43]. Moreover, ANC visits often serve as a platform for mothers to receive information about the significance of vaccinations, the potential risks of non-vaccination, and the available vaccination schedule [44]. Additionally, healthcare settings often integrate maternal and child health services, providing a convenient opportunity for mothers to initiate and complete the vaccination schedule for their children during or immediately after childbirth; particularly, for vaccines that are supposed to be given at birth [45].

Despite the comprehensive analysis conducted in this study, certain limitations remain. Firstly, the data used in this research is based on self-reported information collected in household surveys, which may be subject to recall bias. Additionally, the limitations concern the generalizability of the results. The study focused exclusively on data from Bénin and may not be directly applicable to other contexts or regions with different socio-economic, cultural or health system characteristics.

The results of this study have significant implications for public health policies and interventions aimed at improving immunization programs in Bénin. Targeted interventions for young mothers and people with low levels of education are essential to address disparities in immunization coverage. Strategies should focus on raising awareness and knowledge of the importance of childhood vaccinations through education and awareness programs at the community level. Furthermore, efforts must be made to improve access to immunization services in rural areas, where infrastructure and healthcare delivery systems may be limited. Policymakers and stakeholders should prioritize investment in health infrastructure and human resources to ensure the availability and accessibility of immunization services in all departments.

## Conclusion

This study identified antenatal care visits, health care setting delivery, higher levels of parental school education, and residence in urban areas to be determinants of complete vaccination coverage among children aged 12–59 months in Bénin. Despite the progress made, challenges persist, particularly among young mothers, those with low levels of school education, and in certain geographical regions. This evidence-based analysis provides valuable information for policy-makers, public health practitioners, and stakeholders wishing to strengthen vaccination programs and improve child health outcomes in Bénin.

## Supporting information

S1 Fig Receiver operating characteristic (ROC) curve. (PDF)
