# Supplementary figures and images for: Trends and determinants of complete vaccination coverage among children aged 12–59 months: An analysis of Bénin Demographic and Health Surveys from 1996 to 2018
Source: PLOS Glob Public Health. 2025 Feb 20;5(2):e0004206. doi: 10.1371/journal.pgph.0004206 (PMC11841864; doi:10.1371/journal.pgph.0004206)

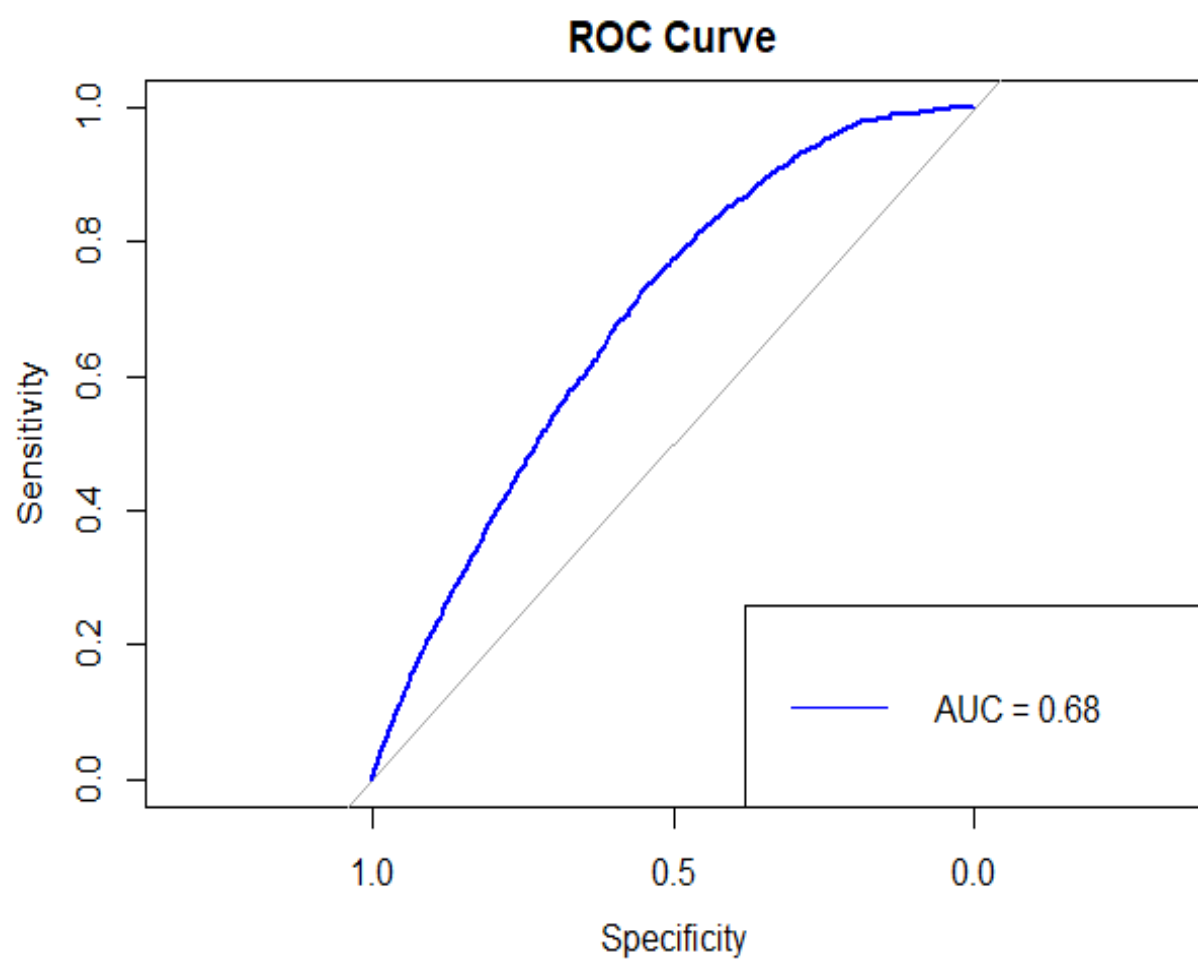

**S1 Fig.** Receiver Operating Characteristic (ROC) curve

Supplement: S1 Fig — (PDF) [file pgph.0004206.s001.pdf]
